# The Proteome and Lipidome of Extracellular Vesicles from *Haemonchus contortus* to Underpin Explorations of Host–Parasite Cross–Talk

**DOI:** 10.3390/ijms241310955

**Published:** 2023-06-30

**Authors:** Tao Wang, Tiana F. Koukoulis, Laura J. Vella, Huaqi Su, Adityas Purnianto, Shuai Nie, Ching-Seng Ang, Guangxu Ma, Pasi K. Korhonen, Aya C. Taki, Nicholas A. Williamson, Gavin E. Reid, Robin B. Gasser

**Affiliations:** 1 Melbourne Veterinary School, Faculty of Science, The University of Melbourne, Parkville, VIC 3010, Australia; guangxu.ma@unimelb.edu.au (G.M.); pasi.korhonen@unimelb.edu.au (P.K.K.); aya.taki@unimelb.edu.au (A.C.T.); 2 The Florey Institute of Neuroscience and Mental Health, The University of Melbourne, Parkville, VIC 3010, Australia; tiana.koukoulis@unimelb.edu.au (T.F.K.); huaqi.su@unimelb.edu.au (H.S.); adityas.purnianto@unimelb.edu.au (A.P.); 3 Department of Surgery, The Royal Melbourne Hospital, The University of Melbourne, Parkville, VIC 3010, Australia; laura.vella@unimelb.edu.au; 4 Department of Biochemistry and Pharmacology, The University of Melbourne, Parkville, VIC 3010, Australia; gavin.reid@unimelb.edu.au; 5 Bio21 Mass Spectrometry and Proteomics Facility, The University of Melbourne, Parkville, VIC 3010, Australia; shuai.nie@unimelb.edu.au (S.N.); ching-seng.ang@unimelb.edu.au (C.-S.A.); nawill@unimelb.edu.au (N.A.W.); 6 Zhejiang Provincial Key Laboratory of Preventive Veterinary Medicine, College of Animal Sciences, Zhejiang University, Hangzhou 310058, China; 7 Bio21 Molecular Science and Biotechnology Institute, School of Chemistry, The University of Melbourne, Parkville, VIC 3010, Australia

**Keywords:** parasitic nematode, *Haemonchus contortus*, proteomics, lipidomics, LC-MS/MS, host-parasite interactions

## Abstract

Many parasitic worms have a major adverse impact on human and animal populations worldwide due to the chronicity of their infections. There is a growing body of evidence indicating that extracellular vesicles (EVs) are intimately involved in modulating (suppressing) inflammatory/immune host responses and parasitism. As one of the most pathogenic nematodes of livestock animals, *Haemonchus contortus* is an ideal model system for EV exploration. Here, employing a multi-step enrichment process (in vitro culture, followed by ultracentrifugation, size exclusion and filtration), we enriched EVs from *H. contortus* and undertook the first comprehensive (qualitative and quantitative) multi-omic investigation of EV proteins and lipids using advanced liquid chromatography–mass spectrometry and informatics methods. We identified and quantified 561 proteins and 446 lipids in EVs and compared these molecules with those of adult worms. We identified unique molecules in EVs, such as proteins linked to lipid transportation and lipid species (i.e., sphingolipids) associated with signalling, indicating the involvement of these molecules in parasite-host cross-talk. This work provides a solid starting point to explore the functional roles of EV-specific proteins and lipids in modulating parasite-host cross-talk, and the prospect of finding ways of disrupting or interrupting this relationship to suppress or eliminate parasite infection.

## 1. Introduction

Parasitic nematodes cause socioeconomically impactful diseases in humans, animals and plants and have a substantial adverse impact on health and agricultural production worldwide [1]. For instance, the roundworm (*Ascaris lumbricoides*), whipworm (*Trichuris trichiura*) and hookworms (*Necator americanus*, *Ancylostoma duodenale* and *A. ceylanicum*) are soil-transmitted helminths that infect ~1.5 billion people globally and affect the poorest and most deprived communities [1]. In the agricultural industry, the trichostrongyloid nematode *Haemonchus contortus* (the barber’s pole worm) and its relatives infect hundreds of millions of sheep and goats worldwide and cause substantial disease and production losses [2]. The control of parasitic nematode infections usually relies on the suppressive or strategic use of anthelmintics. However, excessive and uncontrolled treatment with anthelmintics has led to a drug resistance problem in an increasing number of nematode species worldwide [3,4,5]. This situation has stimulated the search for novel anthelmintic drugs with unique and selective modes of action and effective vaccines [6,7,8].

Deep insights into immune–molecular aspects of parasite-host interactions and parasite–parasite inter-communication could assist in discovering targets for parasitic infection or disease control. Some molecular components of soluble excretory/secretory (ES) products and the external surface (i.e., cuticle) of nematodes have been proposed or shown to modulate the immune responses and/or behaviour of nematodes in host animals [9]. Recently, a growing number of studies (reviewed by [10,11]) also show that parasites produce and release (during infection) extracellular vesicles (EVs), which are essentially (40–150 nm), membrane-bound vesicles containing particular nucleotides (e.g., small RNAs), proteins (e.g., enzymes and toxins) and both membrane-structure-associated and signalling lipids, which are involved in regulating host cell machinery and modulating innate and adaptive immune and inflammatory responses [12,13,14]. Although studies have begun to explore EVs in some parasitic nematode species, such as *Ascaris suum*, *Brugia malayi*, *Heligmosomoides polygyrus*, *Nippostrongylus brasiliensis* and *Trichuris muris* [15,16,17,18,19], there is a significant gap in our understanding of EVs released from the pathogenic nematodes of livestock animals.

Here, we explore the molecular composition of EVs of *H. contortus*—one of the most pathogenic parasitic nematodes of livestock animals (ruminants). This nematode has a direct life cycle, in which first-stage larvae (L1s) hatch from eggs in the environment (pasture) and develop into second-stage (L2s) and then into third-stage larvae (L3s); the host ingests infective L3s, which develop into fourth-stage larvae (L4s) and then into adult male and female worms in the abomasum (stomach), where they feed on blood. *Haemonchus contortus* is a perfect nematode system to undertake this research because extensive genomic, transcriptomic, proteomic and lipidomic resources are now available for this nematode [20,21,22,23,24,25,26,27,28,29] as it is a relative of the metazoan model organism *Caenorhabditis elegans* [30] and shares remarkable conservation in chromosome content, and because of its experimental tractability for fundamental investigations, with implications for drug discovery [6,31,32], vaccine development [33,34] and anthelmintic resistance research [35]. In the present study, we undertook the first comprehensive multi-omic analyses of proteins and lipids in EVs from *H. contortus* enriched using a multistep process (in vitro culture, followed by ultracentrifugation, size exclusion and filtration) combined with advanced liquid chromatography–mass spectrometry and informatic analyses. Our focus was to provide an avenue to understand how the EVs of *H. contortus* are involved in parasite–host interactions and how their cargo is transferred between or among cells, with the goal of opening a door to assessing the utility of EVs as novel therapeutic or vaccine targets.

## 2. Results

### 2.1. Characterisation of H. contortus EVs

**Figure 1:**
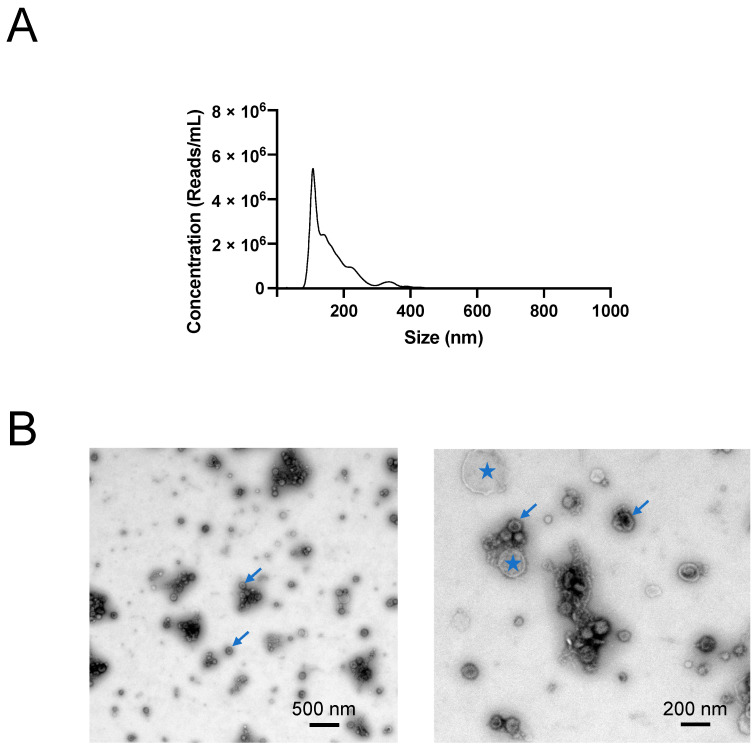
Characterisation of *Haemonchus contortus* EVs. (**A**) Nanoparticle tracking analysis (NTA) revealed the size distribution of size-exclusion chromatography (SEC)-enriched EVs from adult *H. contortus*. The red curves indicate ± standard deviation (SD) for three technical replicates. (**B**) Representative transmission electron microscopy (TEM) of EVs. EVs (blue arrows) were fixed with 1% (*w*/*v*) glutaraldehyde, negatively stained with 2% (*w*/*v*) uranyl acetate and examined by an FEI Tecnai F20 transmission electron microscope. The zoomed-out image (**left**) shows an enriched EV suspension containing limited cellular debris (blue stars) (500 nm scale bar), whereas the zoomed-in image (**right**) reveals round vesicles with membranes (200 nm scale bar).

In accordance with current guidelines (cf. [36]), both nanoparticle tracking analysis (NTA) and transmission electron microscopy (TEM) were performed. NTA revealed an estimated particle size of 164 ± 2 nm (median), with a mean concentration of 3 × 10^11^ ± 4 × 10^10^ particles/mL (Figure 1). TEM images showed the presence of a homogenous population of round, membranous vesicles in the ES products from adult *H. contortus* (Figure 1). Limited cellular debris was observed, highlighting the relative enrichment of EVs in the preparation, achieving 4.6 × 10^8^ particles per protein µg.

### 2.2. The EV Proteome for Adult H. contortus

**Figure 2:**
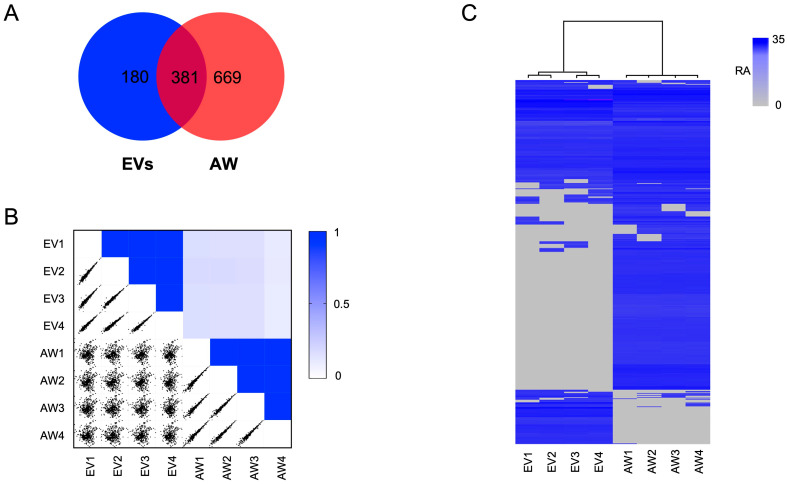
Analyses of the proteomes of EVs and somatic tissues from the adult worm stage (AW) of *Haemonchus contortus*. (**A**) Venn diagram showing the numbers of proteins identified as unique to or shared by the EVs and AW of *H. contortus*. The 180 proteins unique to EVs are marked in Table S1. (**B**) Multi-scatter plots showing the correlation among biological replicates (i.e., EV1–EV4; AW1–AW4) of proteomic changes in the EVs and AW of *H. contortus* upon pairwise comparison. Dark blue represents a high Pearson’s correlation among samples. Each sample was represented by four replicates. (**C**) A heatmap displaying relative protein abundance in EVs or AW of *H. contortus*. Protein abundance (low to high) is shown in grey to blue. RA, relative abundance.

**Figure 3:**
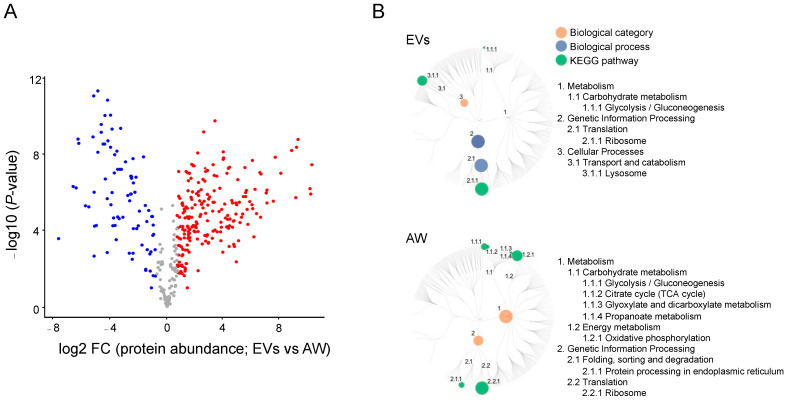
Analyses of differentially expressed proteins in the EVs and adult worm stage (AW) of *Haemonchus contortus*. (**A**) Volcano plots of proteins that were found to be abundant in EVs (blue) and AW (red) of *H. contortus* upon pairwise comparison. Proteins that were not abundant in EVs or AW are indicated in grey. (**B**) Enriched biological processes and associated pathways (KEGG) of differentially expressed proteins in adult worms and EVs from *H. contortus*. Sizes of the dots reflect the number of significantly enriched proteins (cf. Supplementary Table S4).

In total, 561 proteins were identified and quantified from EVs from the adult stage of *H. contortus*. Most of the identified EV proteins (80%, *n* = 451) had significant sequence homology (e-value < 10^−5^) to known proteins, and 20% (*n* = 110) of them did not have any orthologs in current databases and were thus designated as orphan proteins of unknown identity or function (Table S1). Conspicuous among them were molecules such as peptidases (*n* = 74), ribosomal proteins (42), Ras-domain-containing proteins (12), C-type lectins (7) and heat shock proteins (7). A majority of EV proteins (*n* = 381, 68%) were also identified in the somatic proteome of adult *H. contortus*, which contained at least 1050 proteins (Table S2), consistent with previous proteome profiling of adult female and adult male worms of *H. contortus*, with 1677 and 1604 proteins, respectively [27]. A comparison of the numbers of EV and somatic proteins identified herein is given in a Venn diagram (Figure 2A). The largest number of proteins shared between the EV and the somatic proteomes of *H. contortus* were peptidases (*n* = 42) and ribosomal proteins (42), followed by Ras-domain-containing proteins (9). The multi-scatter plot analysis showed consistency in the protein profiles of four replicates within each sample analysed (i.e., EVs and adult worms), whereas the protein profile difference between EVs and somatic tissues was more pronounced (Figure 2B). The hierarchical clustering showed a clear division of the proteomic data set into two distinct groups, corresponding to the EV and somatic proteomes of adult worms (Figure 2C). Pairwise comparison showed substantial differences in protein abundance between EVs and somatic tissues. Specifically, 85 proteins had a higher and 214 proteins had a lower abundance in EVs than in adult worms of *H. contortus* (Figure 3A; Table S3). Nearly half of the 85 proteins were peptidases (45%; 38/85). The lists of proteins identified in EVs and adult worms of *H. contortus* are given in Tables S1 and S2.

KEGG pathway enrichment analysis showed that the differentially abundant EV proteins were predominantly assigned to three biological categories (i.e., genetic information processing, metabolism and cellular processes; Figure 3B). Pathway enrichment for these differentially expressed EV proteins revealed that they were linked predominantly to genetic information processing (*n* = 56) and cellular processes (45), and only a limited number were inferred to be involved in metabolism (29) (Table S4). Genetic information processing and cellular processes were linked exclusively to ribosomes and lysosomes, respectively (Figure 3B). By contrast, proteins with differential abundance in adult worms of *H. contortus* were assigned to the categories of metabolism (*n* = 140) and genetic information processing (104). Within the first category, oxidative phosphorylation (*n* = 59) and glycolysis/gluconeogenesis (37) predominated, followed by citrate cycle (20), glyoxylate and dicarboxylate (13), and propanoate (11) metabolism. For the second category, somatic proteins were inferred to be involved predominantly in ribosome (*n* = 72) and protein processing in the endoplasmic reticulum (32). Extending these analyses, a specific exploration of lipid transportation-related proteins revealed 13 such proteins, including saposin, fatty acid, retinoid-binding proteins, SCP/TAPS proteins and vitellogenin in the EV proteome of adult *H. contortus* (Table 1).

### 2.3. The EV Lipidome for Adult H. contortus

From EVs obtained from adult *H. contortus*, 446 lipid species representing 16 classes and four categories (i.e., glycerolipids, GL; glycerophospholipids, GP; sphingolipids, SP; and sterol lipids, SL) were identified and quantified (see Table 2 and Table S5). Almost 90% of lipid species identified in EVs belonged to the categories GP (*n* = 210) and SP (*n* = 182), while only limited lipid species represented categories GL (*n* = 50) and SL (*n* = 4). In the GP category, glycerophosphoethanolamines (PE) (*n* = 99) and glycerophosphocholines (PC) (*n* = 51) were abundant, whereas in the SP category, ceramide (Cer) (*n* = 86) and sphingomyelin (SM) (*n* = 79) predominated.

For a direct comparison, 638 lipid species from 17 classes and four categories were identified and quantified in the adult stage of *H. contortus* (Table 2 and Table S5), a result that was consistent with previous findings [25]. Unlike lipids identified in EVs, the most commonly identified lipid species were in the categories GP (241), GL (*n* = 211) and SL (159). At the lipid class level, triglycerides (TG) in the GL category represented 30 % (*n* = 189) of the adult worm lipidome, compared with 9% (39 TG species) for EVs. The numbers of identified lipid species shared between EVs and adult worms are listed in Table 2. Most lipid species (*n* = 423; 66%) were common to both EVs and adult worms. EV-specific lipids mainly represented SM (*n* = 7), Cer (7), HexCer (5) and ShexCer (4), whereas TG lipids (*n* =161) were the dominant species in the adult stage of *H. contortus*.

An analysis of fatty acyl compositions showed that lipids in EVs contain high percentages of even- (72.9%) and long-chain fatty acids (>12 carbons; 99.9%) of the total fatty acid composition (Table 3). A detailed analysis revealed that ether-linked lipids were distributed predominantly in the GP category, particularly in the classes PE (*n* = 37), PC (17), LPE (6) and PI (3), whereas the remainder of three ether-linked lipids were all within the GL categories, i.e., DG (2) and TG (1). Most saturated lipid species were in the Cer classes (*n* = 27) PE (20) and PC (9). Notably, only a small proportion of plasmalogen was found in lipids (2%; *n* = 9) of the PE class. Similar to EVs, an analysis of the fatty acyl compositions of the adult worms’ lipidome revealed high percentages of even- (78.3%) and long-chain fatty acids (>12 carbons; 99.5%) in adult worms (Table 3). Lipid classes of PE (*n* = 61), TG (35) and PC (17) represented most of the ether-linked lipids in adult worms, whereas TG (25), PE (22) and LPE (12) were the main classes containing saturated fatty acids.

**Figure 4:**
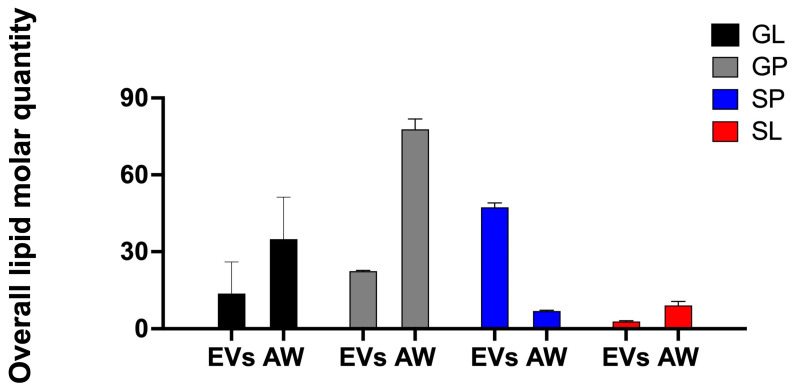
Quantitative differences in glycerophospholipid (GP), glycerolipid (GL), sphingolipid (SP) and sterol lipid (SL) categories between EVs and the adult worm stage (AW) of *Haemonchus contortus*. Semi-quantitative analysis of lipid species, reported as the amount of lipid per μg of protein (pM/μg). Error bars indicate ± standard deviation (SD; three technical replicates).

The quantification of lipid species showed that the GP and SP categories, with large numbers of identified lipid species, contribute markedly to the lipid abundance in the EVs of *H. contortus*. The SP category contained the largest amount of lipids overall, with >45 pM/μg (picomole of lipid per microgram of protein), followed by the GP category, which recorded 22 pM/μg (Figure 4). A detailed appraisal of the SP category revealed that SM (20 pM/μg) and Cer (16 pM/μg) were two abundant classes within this category, characterised by higher abundance of individual SM species (e.g., SM (42:2), SM (34:1), SM (52:3) and SM (42:1)) and Cer species (e.g., Cer (41:1), Cer (43:1), Cer (39:1) and Cer (42:1)). Within the GP category, PC (7 pM/μg), PI (6 pM/μg) and PE (4 pM/μg) were the major classes, being associated with membrane structure. Individual lipid species, including PC (34:1), PC (36:1), PE (36:1), PE (36:2e), PI (36:2) and PI (38:4), containing even-numbered fatty acyl chain 16:0, 18:0, 18:1, 18:2 or 20:4, predominated (Table S5).

Compared with the EV lipidome, the adult worms of *H. contortus* contained a larger amount of lipids overall, particularly those in the GL and GP categories. The largest amount of lipids overall was measured in the GP category, with >77 pM/μg, followed by categories GL (47 pM/μg), SP and SL (both < 10 pM/μg) (Figure 4). The most abundant lipid class recorded was the energy-storage-related lipid TG (45 pM/μg), followed by PC (33 pM/μg), PI (27 pM/μg) and CE (9 pM/μg). Further analysis of individual lipid species revealed TG lipids, with C16 and C18 fatty acyl chains (e.g., 16:0, 16:1, 18:0, 18:1, 18:2 and 18:3) predominating and many of them (*n* = 19) being abundant (>1 pM/μg) (Table S6). There was no significant difference in lipid categories between EVs and AW.

## 3. Discussion

Using ultracentrifugation- and size-exclusion chromatography-based approaches, we isolated and enriched EVs from the ES products from adult female and male worms of *H. contortus*. The presence of EVs was verified by NTA and TEM, and the morphology and morphometrics of these EVs were consistent with those observed previously [37]. Following this appraisal, we characterised the proteins in purified *H. contortus* EVs and showed consistency in these proteins with previous studies of other nematodes. For instance, actin (*n* = 1), M13 metallopeptidases (2), heat-shock protein 70 (3), transthyretin-like family proteins (2) and SCP/TAPS proteins (6) have been commonly reported in EV proteomes of parasitic nematodes including *A. suum*, *B. malayi*, *He. polygyrus*, *N. brasiliensis* and *T. muris* (review by [11]), suggesting some conservation in secreted proteins at the functional level among parasitic nematode species. Further investigation of the potential utility of these molecules as biomarkers for the specific detection or diagnosis of nematode infection in animals is warranted.

The identification of tetraspanin (i.e., HCON_00008900) here in EVs of *H. contortus* (but not adult worms) is noteworthy as this molecule is widely regarded as an EV marker due to the presence of orthologs on the surfaces of EVs from various parasitic worms, including trematodes, such as *Fasciola hepatica*, *Schistosoma mansoni* and *Opisthorchis viverrini* (see [38,39,40]). While parasitic trematodes excrete/secrete or shed EVs enriched with tetraspanins [38,39,40], these proteins appear not to be abundant on the surface of the EVs of nematodes. For instance, only one member of the tetraspanin family was identified in EVs from the parasitic nematode *He. polygyrus* [41]. Although the reason for tetraspanins not being enriched in nematode EVs remains unclear, these molecules are known to be involved in EV membrane formation [42]. It is likely that nematode EVs originate from their alimentary and/or genital tract(s) or the secretory system rather than from the cuticle, as opposed to trematode EVs which originate, to a large extent, from the tegument, in addition to the digestive and genetic tracts [43].

Almost 40% (*n* = 265) of the proteins identified in *H. contortus* EVs were either unique or abundant compared with proteins from adult worms (Table S3). It is likely that some of these EV proteins, such as SCP/TAPS proteins (*n* = 6) and C-type lectins (7), play key roles in host–parasite interactions, including invasion and immunosuppression/modulation [44,45]. SCP/TAPS proteins are present in nearly every nematode species studied to date; the numbers of these proteins reported to date vary from a few (e.g., *B. malayi* and *A. suum*) to more than a hundred (*A. caninum*) [46]. A previous genomic study [24] revealed 84 SCP/TAPS proteins (22 double and 62 single SCP-like domain molecules) encoded in the genome of *H. contortus*. Among them, 43 SCP/TAPS protein-coding genes were transcribed at high levels in parasitic (i.e., L4 and adult) stages of *H. contortus* [24]. Although an early proteomic analysis of adult *H. contortus* ES products using a two-dimensional gel-based approach (in the absence of genomic/transcriptomic resources at the time) identified only two SCP/TAPS proteins (Hc24, containing a single SCP-like domain, and Hc40, containing two such domains) [47]. In contrast, in a deep proteomic profiling study of the secretome of *H. contortus*, we identified 44 SCP/TAPS proteins, 29 (66.7%) of which were shown to be abundant in the ES proteins collected from L4 and female adult stages [29]. Here, we identified six particular SCP/TAPS proteins in EVs (Table S1) that were within the same group of 29 proteins identified in the secretomes from parasitic stages of *H. contortus* (cf. [29]), indicating their likely relevance in host–parasite interplay, including immunomodulatory functions; this finding warrants further research on the functional roles of these six proteins. Although the function of these molecules is presently unknown, previous work has inferred their essential roles in parasite–host crosstalk to promote nematode survival and development within the host animal (reviewed by [46]). Recent studies of parasitic worms of the genera *Heligmosomoides* and *Schistosoma* [48,49] have shown that SCP/TAPS proteins contain a structurally conserved lipid-binding cavity (in an alpha-beta-alpha sandwich structure) that might be involved in lipid-associated signalling in their host animals in order to modulate (potentially suppress) immune responses. The discovery of multiple proteins (*n* = 13) linked to lipid transportation (i.e., saposin, fatty acids, retinoid-binding proteins, SCP/TAPS proteins and vitellogenin) and of unique signalling lipids of the SP category in the EVs of *H. contortus* warrants future research to evaluate the functional roles of these six proteins in parasitism.

Although lipids are key EV components, our current understanding of their composition and contribution to function is scant. Although the lipid composition of EVs varies, depending on the cell type from which they originate, their physiological state and their microenvironment, as membrane-bound structures, EVs from mammalian cells are known to be rich in GP (e.g., PS), CE and SP (e.g., SM) (reviewed by [50]). Currently, the lipid composition and abundance of EVs from parasites are largely unexplored. Thus far, the only lipidomic analysis of EVs of a parasitic nematode called *He. polygyrus* (an intestinal parasite of rodents) revealed a unique lipid profile and a very high content of PE plasmalogens (47% of overall lipid abundance) and small amounts of CE (7%) and SM (3%) lipids [41]. The findings of the latter study suggested that the high level of ether-linked PE was related to the rigidity of vesicle membranes and compensated for the small amounts of CE and SM [41]. Interestingly, our result showed a distinct lipid content profile for *H. contortus* EVs. Significant amounts of membrane structure-related SL (SM and Cer) and GP (PC, PE and PI, LPC) lipid categories and a low abundance of PE plasmalogens (2% of overall lipid abundance) were observed in the lipidome of EVs from adult *H. contortus*. Whether the high expression levels of SL and GP are compensated for by the low level of plasmalogens and/or relate to the stability and fluidity of membrane-enriched EVs requires further investigation.

The focus of most studies of EVs from parasitic worms has been on proteins and nucleic acids (small RNAs) (reviewed by [11]). However, it is likely that lipids play critical roles in EVs, particularly relating to structural components of membranes and/or as intra- and intercellular signalling molecules [51,52]. Some evidence indicates that cell-signalling-related lipids (i.e., PC, PI and LPC) are abundant in some of these worms and likely play multifaceted roles in host–parasite interactions to modulate host immune responses to enable parasite survival. For instance, an early study showed that a PC-containing product (i.e., ES-62) excreted or secreted by *Acanthocheilonema viteae* (a filarioid nematode) can disrupt B cell function by targeting signalling pathways linked to cell proliferation [53]. In other work, LPC and LPS species from the blood fluke *S. mansoni* were shown to stimulate Toll-like receptor-2-dependent pathways in the host immune system, resulting in reduced host immunomodulation characterised by reduced eosinophil activation and cytokine production [54,55]. Thus, it is likely that some lipid species play a presently under-appreciated role in host–parasite interactions. Notably, the presence of multiple lipid transportation-related proteins (*n* = 13) (i.e., saposin, fatty acid, retinoid-binding proteins, SCP/TAPS proteins and vitellogenin) that enable the transfer of signal lipids (e.g., phospholipids) between membranes [56] further support the likelihood of lipids being actively involved in host–parasite interplay.

## 4. Materials and Methods

### 4.1. Parasite Stages

*Haemonchus contortus* was produced in Merino lambs (10 months of age; Victoria, Australia) maintained under helminth-free conditions. Sheep were inoculated by gavage with 10,000 infective L3s of *H. contortus*. Adult worms were produced as described previously [57]. In brief, these worms were collected from the abomasa of infected lambs, following euthanasia by intravenous injection of pentobarbitone sodium (Virbac, Carros Cedex, France) 28 days after infection with L3s. Animal ethics approval (no. 1714374) was by The University of Melbourne.

### 4.2. Preparation of ES Products

ES products from *H. contortus* were collected using an established protocol [29], following the accepted guidelines for parasitic helminths [36]. Briefly, adult worms were washed extensively in pre-warmed (37 °C) physiological saline and RPMI 1640 containing 10 mM L-glutamine (BioWhittaker; cat no. 12-702F; Lonza, Basel, Switzerland), respectively. They were then suspended in the latter medium containing 100 IU/mL of penicillin, 100 μg/mL of streptomycin and 2.5 μg/mL of amphotericin (fungizone, antibiotic-antimycotic; cat no. 15240-062; Gibco, Waltham, MA, USA) in tissue culture flasks (175 cm^2^, vented cap, BD Falcon, Schaffhausen, Switzerland) at a concentration of 3 adult worms per ml and incubated at 37 °C in 10% (*v*/*v*) CO_2_. In total, ~1000 adults were cultured to obtain sufficient ES products and associated EVs. To eliminate host contamination, the culture medium was discarded after the first 3 h of culture and replaced with a fresh medium (37 °C, 10% (*v*/*v*) CO_2_). Subsequently, the culture medium was replaced and collected every 24 h for 2 days. The medium batches were pooled for subsequent EV isolation. The viability of the worms was assessed every 12 h. The collected culture medium was processed by differential centrifugation to remove intact worms (480× *g* for 5 min) and cell debris (2000× *g* for 10 min) at 4 °C and then stored at −80 °C until EV isolation.

### 4.3. EV Enrichment

The procedure for the enrichment of EVs from *H. contortus* was inspired by a method used for bacteria [58] and conducted according to accepted guidelines for parasitic helminths [36]. First, ES products were centrifuged at 10,000× *g* (Fiberlite F9-4 X 1000y fixed angle rotor, Sorval RC-6+ Superspeed centrifuge, Thermo Fisher Scientific, Denver, CO, USA) for 30 min at 4 °C. The supernatant was then filtered using a vacuum filter (0.22 μm aperture) and then concentrated to ~300 mL using centrifugal ultrafiltration (Centricon^®^ Plus-70 10 kDa MWCO spin concentrators; Amicon, Merck Millipore, Cleveland, OH, USA) at 4 °C. The concentrated supernatant was then centrifuged at 129,000× *g* (Fibrelite F37L8-100 fixed angle rotor, Sorval WX 100 Ultracentrifuge, Thermo Fisher Scientific, USA) for 3 h at 4 °C to recover crude EVs. These EVs were resuspended in phosphate-buffered saline (PBS; pH 7.4) containing a complete protease inhibitor cocktail (1:50 dilution; Merck, Søborg, Denmark) and purified using a size-exclusion chromatography-based approach [59]. After rinsing the column with PBS (2 × 70 mL), EVs were suspended in PBS (20 mL), loaded onto a qEV10/35 nm column (Izon Science, Christchurch, New Zealand), eluted (20 mL) and then concentrated using an Amicon Ultra-4 10 kDa centrifugal filter device (Merck Millipore, USA). A complete protease inhibitor cocktail and phosSTOP phosphatase inhibitor cocktail (1:50 and 1:10, respectively; Merck, Denmark) were added to the concentrated EV elution. An aliquot (15 μL) of this elution was sonicated using an ice water bath sonicator for 20 min and assessed using a bicinchoninic acid (BCA) assay according to the manufacturer’s protocol (Thermo Fisher Scientific, USA). In total, 1055 μL of EVs at a protein concentration of 647 μg/mL were aliquoted and stored at 4 °C overnight for nanoparticle tracking analysis (NTA; Section 4.4) in 1% glutaraldehyde at 4 °C for transmission electron microscopy (Section 4.5) or at −80 °C for subsequent proteomic and lipidomic analyses (Section 4.6, Section 4.7, Section 4.8 and Section 4.9).

### 4.4. NTA

The size distribution and concentration of isolated vesicles were determined using NTA. The samples were diluted 1:1000 in filtered phosphate-buffered saline (PBS; Thermo Fisher Scientific, USA) prior to infusion into a NanoSight NS300 (Malvern Panalytical, Malvern, UK) flow-cell using a 1 mL syringe at a flow rate of 40 µL/s. Five 30 s long videos were recorded. NanoSight NS300 NTA software was used to analyse both the size and concentration of particles. Three technical replicates were included.

### 4.5. TEM

For TEM, purified EVs (5 μL) were fixed in electron microscopy-grade 1% (*v*/*v*) glutaraldehyde, absorbed onto neutralised 300-mesh carbon-coated formvar copper grids (ProSciTech, Kirwan, Queensland, Australia) for 1 min. The grids were washed twice with MilliQ water and then stained with 2% (*w*/*v*) saturated aqueous uranyl acetate for 1 min. Electron microscopy was performed using a Tecnai G2 F20 microscope (FEI, Eindhoven, The Netherlands) operating at 300 kV in the Ian Holmes Imaging Center at Bio21 Institute, University of Melbourne.

### 4.6. Extraction of Proteins and Proteomic Analysis by LC-MS/MS

Samples of enriched EVs (four technical replicates each containing 50 μg of protein) were sonicated (using a 160TD Ultrasonic Cleaner; Soniclean, Dudley Park, South Australia, Australia) in a lysis buffer (8 M urea in 100 mM triethyl ammonium bicarbonate, pH 8.5) at 18 °C for 20 min. In addition, total somatic protein samples from adult worms (male and female) were prepared as described previously [27]. In brief, ~20 pooled, freshly collected worms of each sex were subjected to three freeze (−196 °C)–thaw (37 °C) cycles and then ultrasonicated (20 kHz) using a BioRuptor (10 cycles: 30 s on/30 s off) on ice in separate low protein-binding Eppendorf tubes (1.5 mL; Merck, Denmark), each containing 300 μL of lysis buffer (8 M urea in 100 mM triethyl ammonium bicarbonate, pH 8.5) and supplemented with protease inhibitor cocktail set I (1:100 dilution; Merck, Denmark). The protein amount in each sample was measured using a BCA assay. In-solution digestion was performed [60] and samples were reduced with 10 mM Tris (2-carboxyethyl) phosphine (TCEP) at 55 °C for 45 min, alkylated with 55 mM iodoacetamide in the dark at 22 °C for 30 min and double-digested with a Lys-C/trypsin mix (Promega, Madison, WI, USA) at 37 °C for 16 h (4 h for Lys-C and 12 h for trypsin digestion), as recommended by the manufacturer. The tryptic samples were acidified with 1.0% (*v*/*v*) formic acid and purified using Oasis HLB cartridges (Waters, Milford, MA, USA). Then, samples were freeze-dried and then re-suspended in aqueous 2% *w*/*v* acetonitrile and 0.05% *w*/*v* trifluoroacetic acid (TFA) prior to LC-MS/MS analysis.

Tryptic peptides were analysed using an Exploris 480 Orbitrap mass spectrometer (Thermo Fisher Scientific, USA) equipped with an Acclaim Pepmap nano-trap column (Dinoex-C18, 100 Å, 75 µm × 2 cm) and an Acclaim Pepmap RSLC analytical column (Dinoex-C18, 100 Å, 75 µm × 50 cm). Tryptic peptides were injected into the enrichment column at an isocratic flow of 5 µL/min of 2% (*v*/*v*) CH_3_CN containing 0.05% (*v*/*v*) trifluoroacetic acid (TFA) for 6 min, applied before the enrichment column was switched in-line with the analytical column. Solvent A was (*v*/*v*) 0.1% formic acid, 95% H_2_O and 5% dimethyl sulfoxide and solvent B was (*v*/*v*) 0.1% formic acid, 95% acetonitrile and 5% dimethyl sulfoxide. The gradient was at 300 nL/min from (i) 0–6 min at 3% B; (ii) 6–95 min, 3–20% B; (iii) 95–105 min, 20–40% B; (iv) 105–110 min, 40–80% B; (v) 110–115 min, 80–80% B; and (vi) 115–117 min 85–3% and equilibrated at 3% B for 10 min before injecting the next sample. The Exploris 480 Orbitrap mass spectrometer was operated in the data-dependent mode, whereby full MS1 spectra were acquired in a positive mode (spray voltage of 1.9 kV; source temperature of 275 °C), with 120,000 mass resolving power (at *m*/*z* 200), an AGC target of 3 × 10^6^ and a maximum IT time of 25 ms. The “top 3 s” acquisition method was used and peptide ions with charge states of 2–6 and intensity thresholds of ≥5 × 10^3^ were isolated for MS/MS. The isolation window was set at 1.2 *m*/*z* and precursors were fragmented using higher energy collisional dissociation (HCD) at a normalised collision energy of 30, resolution of 15,000, normalized AGC target of 75% and with automated IT time selected. Dynamic exclusion was set at 30 s.

### 4.7. Protein Identification and Quantification

The proteome predicted from the genome *H. contortus* [20] was annotated using NCBI non-redundant protein database (NR) [61]. Raw data were processed using MaxQuant [62]. Enzyme specificity was Trypsin/P with a maximum of 2 missed cleavages, and the search parameters were as follows: a precursor tolerance of 20 parts per million (ppm) for the “first search” and 4.5 ppm for the “main search”; an MS/MS tolerance of 20 ppm; and fixed modifications for carbamido-methylation of cysteine (+57 Da) and methionine oxidation (+16 Da). Results were accepted based on a false discovery rate (FDR) of <0.01 at both the peptide and protein levels. Proteins were quantified using the LFQ value from MaxQuant employing default settings. Only proteins of ≥2 peptides and identified in ≥2 biological replicates were accepted. The mass spectrometry proteomic data (identifier PXD041918) are accessible via the PRIDE archive of the ProteomeXchange Consortium [63].

### 4.8. Extraction of Lipids and Lipidomic Analysis by HPLC-MS/MS

Total lipids in EVs and adult worms were extracted using an established monophasic extraction protocol [64]. Briefly, samples of EVs (50 μg of protein) or freshly collected worms (8 mg body weight) were individually transferred to Eppendorf tubes (1.5 mL), each containing 20 μL or 200 μL of ice-cold 40% (*v*/*v*) methanol with 10 μL of an isotope labelled as an internal lipid standards solution (330710X, Mouse SPLASH^®^ LIPIDOMIX, Merck, USA). Samples (four replicates representing EVs and adult worms of each) were homogenised with 100 μL of 0.5 mm zirconium oxide beads (ZROB05, Next Advance, Troy, NY, USA) in a blender (Bead Bullet, Next Advance, USA) for 3 cycles (30 sec on at speed 8 and 30 s off on ice). Then, 700 μL of 0.74/1/2 (*v*/*v*/*v*) water/chloroform/methanol was added to each homogenised sample. The tubes were vortexed for 60 s and incubated on a ThermoMixer (Eppendorf, Hamburg, Germany) at 1000× *g* for 30 min at 22 °C and then centrifuged at 14,000× *g* for 15 min. The supernatant was then transferred to another tube and 400 μL of 1/2 (*v*/*v*) chloroform/methanol was added, vortexed (10 s) and centrifuged, as before. The supernatant was collected and combined with the supernatant from the first extraction. Individual combined lipid samples were then dried in a SpeedVac, and each lipid pellet was re-suspended in 100 μL of 4/2/1 (*v*/*v*/*v*) isopropanol/methanol/chloroform containing 0.01% butylated hydroxytoluene (BHT) prior to analysis. Blank tubes with water (included as controls) were processed in the same manner.

Samples were analysed by ultrahigh performance liquid chromatography (UHPLC) employing a Vanquish UHPLC coupled to an Orbitrap Fusion Lumos mass spectrometer (Thermo Fisher Scientific, USA), with separate runs in positive and negative ionisation mode polarities. Solvent A was 6/4 (*v*/*v*) acetonitrile/water with 5 mM medronic acid and solvent B was 9/1 (*v*/*v*) isopropanol/acetonitrile; both solvents A and B contained 10 mM of ammonium acetate. Each sample (10 μL) was injected into an Acquity UPLC HSS T3 C18 column (1 × 150 mm, 1.8 µm; Waters, USA) at 50 °C at a flow rate of 80 μL/min for 3 min using 3% solvent B. During separation, the percentage of solvent B was increased from 3% to 70% over 5 min and from 70% to 99% over 16 min. Subsequently, the percentage of solvent B was maintained at 99% for 3 min. Finally, the percentage of solvent B was decreased to 3% over 0.1 min and maintained for 3.9 min.

All MS experiments were performed using a heated electrospray ionization (HESI) source. The spray voltages were 3.5 kV in positive ionisation mode and 3.0 kV in negative ionisation mode. In both polarities, the flow rates of sheath, auxiliary and sweep gases were 25, 5 and 0 “arbitrary” unit(s), respectively. The ion transfer tube and vaporizer temperatures were maintained at 300 °C and 150 °C, respectively, and the S-Lens RF level was set at 50%. In the positive ionisation mode from 3 to 24 min, a top-speed data-dependent scan with a cycle time of 1 s was used. Within each cycle, full-scan MS-spectra were acquired firstly in the Orbitrap at a mass resolving power of 120,000 (at *m*/*z* 200) across an *m*/*z* range of 300–2000 using quadrupole isolation, an automatic gain control (AGC) target of 4 × 10^5^ and a maximum injection time of 50 milliseconds, followed by HCD-MS/MS at a mass resolving power of 15,000, a normalised collision energy (NCE) of 27% in positive ionisation mode and 30% in negative ionisation mode, an *m*/*z* isolation window of 1, a maximum injection time of 35 ms and an AGC target of 5 × 10^4^. For the improved structural characterisation of glycerophosphocholine (PC) lipid cations, a data-dependent product ion (*m*/*z* 184.0733)-triggered collision-induced dissociation (CID)-MS/MS scan was performed in the cycle using a q-value of 0.25 and a NCE of 30%, with other settings being the same as that for HCD-MS/MS. For the improved structural characterisation of TG lipid cations, the (fatty acid + NH_3_) neutral loss product ions observed by HCD-MS/MS were used to trigger the acquisition of top-3 data-dependent CID-MS^3^ scans in the cycle using a q-value of 0.25 and a NCE of 30%, with other settings being the same as those for HCD-MS/MS.

### 4.9. Identification and Quantification of Lipids and Statistical Analysis

LC-MS/MS data were searched through MS Dial 4.80. The mass accuracy settings were 0.005 Da and 0.025 Da for MS1 and MS2, respectively. The minimum peak height was 50,000 and the mass slice width was 0.05 Da. The identification score cut off was 80%. Post-identification was conducted with a text file containing the name and m/z of each standard in the SPLASH^®^ LIPIDOMIX^®^ Mass Spec Standard (330710X, Avanti Polar Lipids, Alabaster, AL, USA). In positive ionisation mode, [M+H]^+^, [M+NH_4_]^+^ and [M+H-H_2_O]^+^ were selected as ion forms. In negative ionisation mode, [M-H]^−^ and [M+CH_3_COO]^−^ were selected as ion forms. All lipid classes available were selected for the search. The retention time tolerance for alignment was 0.1 min. Lipids with maximum intensity less than 5-fold of the average intensity in blank were removed. All other settings were default. All lipid LC-MS features were manually inspected and re-integrated when needed. Four types of lipids, (1) lipids with only sum composition except for SM, (2) lipid identifications due to peak tailing, (3) retention time outliers within each lipid class and (4) LPA and PA artifacts generated by in-source fragmentation of LPS and PS, were also removed. The shorthand notation used for lipid classification and structural representation followed the nomenclature proposed previously [65].

Relative quantification of lipid species was achieved by normalisation of the LC peak areas of identified lipids against peak areas and concentrations of the corresponding internal lipid standards from the same lipid class. Finally, the lipid species at the class, subclass or molecular species levels were normalised to either the total lipid concentration (i.e., mol% total lipid) or total lipid class concentration (i.e., mol% total lipid class). For lipid classes or sub-classes without correspondent stable isotope-labelled lipid internal standards, the LC peak areas of individual molecular species within these classes were normalised as follows: MG species against DG (18:1D7_15:0); CL against PI (18:1D7_15:0); LPG against PG (18:1D7_15:0), LPA against PA (18:1D7_15:0), LPS against PS (18:1D7_15:0); and Hex1Cer and Cer against SM (d36:2D9). Given that the commercial availability of some stable isotope-labelled lipid standards is limited, some of the identified lipids were normalised against a standard from a different class or sub-class, and no attempts were made to quantitatively correct for the different ESI responses of individual lipids due to concentration, acyl chain length, degree of unsaturation or matrix effects caused by differences in chromatographic retention times compared with the relevant standards. The results reported here are, therefore, for relative quantification and should not be considered to reflect the absolute concentrations of each lipid or lipid sub-class. The mass spectrometry lipidomic data (identifier PXD043320) are accessible via the PRIDE archive of the ProteomeXchange Consortium [63].

### 4.10. Bioinformatic Analyses of Data Sets

For proteomic data, the UniProt repository was used for protein annotation (according to cellular compartment, subcellular location, transmembrane region and/or molecular function). Molecular functions of proteins were assigned according to Gene Ontology (GO) using the program InterProScan [66]. Venn diagrams were drawn using the VennDiagram package in R. Sequence homology searches were conducted using BLASTP (https://blast.ncbi.nlm.nih.gov/Blast.cgi?PAGE=Proteins, accessed on 8 March 2023). Volcano plot analysis was employed to assess differential protein expression using Perseus software (v.1.6.1.1) [67], with the Permutation-based false discovery rate (FDR) and fold change (FC) set at Q ≤ 0.01 and >2, respectively. Biological functions were assigned to differentially expressed proteins using the Kyoto Encyclopedia of Genes and Genomes (KEGG) database [68]. KEGG pathway annotation was conducted employing KEGG BLASTP hits (E-value: <10^−5^) and corresponding KEGG Orthology (KO) terms [69]. KO terms were then assigned to KEGG pathways and KEGG BRITE orthologous protein families by mapping these terms to the KEGG Orthology-Based Annotation System (KOBAS) database [70]. Enriched KEGG pathways were identified using a cut-off of *p* < 0.01 (Fisher’s exact test). KEGG functional enrichments of differentially expressed proteins were integrated and displayed using the program FuncTree [71].

For lipidomic data, a one-way ANOVA post hoc test was performed for multiple group comparisons by using GraphPad Prism 8.4.2 software (GraphPad, La Jolla, CA, USA). Error bars represent the standard deviation (SD). Statistical significance was set at adjusted *p* < 0.05.

## 5. Conclusions

In this study, we present significant insights into macromolecules excreted/secreted as EVs by *H. contortus*. The detection and characterisation of proteins and lipids present in EVs should have important implications for understanding the mechanisms by which the parasite creates a favourable host environment for its survival, which could inform new ways of combating *H. contortus* infection and haemonchosis in animals. Our findings raise the prospect of such molecules within EVs as therapeutic targets or biomarkers and emphasise the importance of conducting further research into the intricate interactions between *H. contortus* and its host animals. We propose that host (e.g., sheep) organoids will assist in exploring the involvement of individual or grouped EV molecules in regulating or altering molecular processes or mechanisms in host cells. Extending this work to undertake detailed comparative studies of EVs and their functional relevance in other parasitic worms is likely to deepen our knowledge and understanding of how such worms modulate (e.g., suppress) inflammatory and immune responses in host animals.

## Figures and Tables

**Table 1:** List of lipid transportation-related proteins identified in EVs of *Haemonchus contortus*.

Protein	Accession Code
Saposin	HCON_00047370; HCON_00047380
Nematode fatty acid retinoid-binding proteins	HCON_00092780; HCON_00092800; HCON_00092810
Fatty acid-binding proteins	HCON_00169150
SCP/TAPS proteins	HCON_00100510; HCON_00137280; HCON_00140240; HCON_00146300; HCON_00148510; HCON_00192680
Vitellogenin	HCON_00188470

**Table 2:** Numbers of lipid species identified in EVs and in adult worms of *Haemonchus contortus*.

Lipid Category/Class	EVs	Adult Worms	Shared by EVs and Adult Worms	Total Number
Glycerolipids				
DG	11	22	11	22
TG	39	189	39	189
Glycerophospholipids				
PC	51	54	51	54
PE	99	106	99	106
PG	2	3	2	3
PI	12	18	12	18
PS	6	6	6	6
CL	5	12	5	12
LPC	16	18	16	18
LPE	18	19	18	19
LPI	0	2	0	2
LPS	1	3	1	3
Sphingolipids				
SM	70	63	63	79
Cer	80	73	73	80
HexCer	26	21	21	26
ShexCer	6	2	2	6
Sterol lipids				
CE	4	4	4	4
In total	446	615	423	638

Abbreviations: MG, monoradylglycerols; DG, diradylglycerols; TG, triradylglycerols; PC, glycerophosphocholines; PE, glycerophosphoethanolamines; PG, glycerophosphoglycerols; PI, glycerophosphoinositols; PS, glycerophosphoserines; CL, cardiolipins; SM, sphingomyelins; Cer, ceramide; HexCer, hexosylceramide; ShexCer, sulfatides hexosylceramide; CE, cholesteryl ester. Note: For LPC, LPE, LPI and LPS, prefix “L” was added for each lysoglycerophospholipid class.

**Table 3:** Fatty acyl compositions of lipid species in the lipidome of EVs and adult worms of *Haemonchus contortus*.

Lipid Category (EVs/Adult Worms)	Saturated (%)		Unsaturated (%)		Odd-Chain FA (%)	Even-Chain FA (%)	Total No. of FA
	Medium-Chain FA	Long-Chain FA	Medium-Chain FA	Long-Chain FA			
Glycerolipids	ND/0.4	7.8/19.5	ND/ND	8.2/25.8	2.3/6.7	13.7/39.0	138/651
Glycerophospholipids	0.1/0.1	21.1/13.5	ND/ND	25.9/20.3	8.7/5.7	38.4/28.2	406/482
Sphingolipids	ND/ND	21.2/11.4	ND/ND	15.2/8.7	16.1/9.3	20.3/10.8	314/286
Sterol lipids	ND/ND	ND/ND	ND/ND	0.5/0.3	ND/ND	0.5/0.3	4/4
Total	0.1/0.5	50.1/44.4	ND/ND	49.8/55.1	27.1/21.7	72.9/78.3	862/1423

FA, fatty acyl; ND, not detected; medium-chain FAs contain 6–12 carbons; long-chain FAs contain >12 carbons.

## Data Availability

The mass spectrometry proteomic (identifier PXD041918) and lipidomic (identifier PXD043320) data are accessible via the PRIDE archive of the ProteomeXchange Consortium.

## Supplementary Materials

The following supporting information can be downloaded at https://www.mdpi.com/article/10.3390/ijms241310955/s1.
